# The Contributions of Segmental and Suprasegmental Information in Reading Chinese Characters Aloud

**DOI:** 10.1371/journal.pone.0142060

**Published:** 2015-11-09

**Authors:** Min Wang, Chuchu Li, Candise Y. Lin

**Affiliations:** 1 Department of Human Development and Quantitative Methodology, University of Maryland, College Park, Maryland, United States of America; 2 Department of Psychology and Program in Hearing and Communication Neuroscience, University of Southern California, Los Angeles, California, United States of America; Leiden University, NETHERLANDS

## Abstract

The Chinese writing system provides an excellent case for testing the contribution of segmental and suprasegmental information in reading words aloud within the same language. In logographic Chinese characters, neither segmental nor tonal information is explicitly represented, whereas in Pinyin, an alphabetic transcription of the character, both are explicitly represented. Two primed naming experiments were conducted in which the targets were always written characters. When logographic characters served as the primes (Experiment 1), syllable segmental and tonal information appeared to be represented and encoded as an integral unit which in turn facilitated target character naming. When Pinyin served as the primes (Experiment 2), the explicit phonetic representation facilitated encoding of both segmental and suprasegmental information, but with later access to suprasegmental information. In addition, Chinese speakers were faster to name characters than Pinyin in a simple naming task (Experiment 3), suggesting that Pinyin may be read via a phonological assembly route, whereas characters may be read via a lexical route. Taken together, our findings point to the need to consider the contributions of both segmental and suprasegmental information and the time course in the well-established models for reading aloud, as well as the cognitive mechanisms underlying the reading aloud of logographic characters versus alphabetic Pinyin script.

## Introduction

The importance of phonological information in reading has been studied extensively in the literature. Reading, broadly defined, encompasses both visual word recognition (silent reading) and reading aloud. The current study focused on phonological encoding and processing in reading aloud. Studies have shown that phonological information is activated in both visual word recognition and reading aloud in alphabetic writing systems [1,2]. However, the Chinese writing system is logographic. There is no explicit representation of phonological information in the orthography; for example, the character 马, meaning horse, is pronounced as *ma3* (the number 3 here denotes the tone). Note that none of the components of this character refers to the phonological constituents /m/, /ɑ/ or tone 3 (T3). Although some characters contain phonetic radicals, which encode or specify the sound of the character (e.g., 蚂 /ma3/ *ant* deriving its sound from its phonetic radical 马), linguistic analyses have shown that a phonetic radical could only accurately predict the pronunciation of a character 23–26% of the time when tone is taken into account [3,4]. Given the lack of grapheme-phoneme mapping in Chinese characters, the mechanism and time course underlying the activation of segmental and tonal information in visual character recognition and reading characters aloud has been an interesting and important question for discussion over the past two decades (e.g., ref [5–7]).

Phonology encompasses segmental and suprasegmental information. Segments consist of vowels and consonants while suprasegmental features are speech attributes that accompany consonants and vowels but which are not limited to single sounds and often extend over syllables, words, or phrases [8]. Both segmental and suprasegmental information provide useful information in spoken word recognition. For example, the pronunciations of the English words *pie* and *buy* differ only in their initial phoneme segment (/p/ vs. /b/), yet their meanings and syntactic categories are completely different. In the spoken word *record*, when stress is placed on the first syllable *RECord*, it is a noun meaning *an account of facts*. When stress is placed on the second syllable *reCORD*, it becomes a verb and means *to set down in writing*. With regard to visual word recognition and reading aloud, previous research has focused mainly on the importance of segmental phonology. How different types of phonological constituents function in reading in general (e.g., segments vs. suprasegments) has received relatively less attention. Ashby and Clifton showed that stress information is indeed represented and activated in silent reading of English words [9]. This eye-tracking study showed that the number of stressed syllables in a word had an impact on word recognition in silent reading. Readers spent more time on words that contained two stressed syllables than those with one stressed syllable, controlling for factors such as word length and frequency. Similar to lexical stress in English, lexical tone is part of the suprasegmental phonology in Mandarin Chinese.

One of the most influential models in reading literature is the dual-route model of reading aloud proposed by Colheart and colleagues [10–12]. This model claims that there are two distinct pathways to reading aloud of written words. The first route provides a direct linkage from visual input to a word’s phonology. It can go either directly from orthography to phonology, or go through semantic system to phonology. This route is referred to as the addressed, or lexical route. The second route converts graphemes into phonemes, either one by one or in strings, which are used to access the word’s phonology. The second route is referred to as the assembled, or non-lexical route. The key difference between the two routes is that the assembled route uses the assembly of the word’s phonology as an intermediate step in the process, whereas the addressed route is the direct look-up of an address.

The dual-route reading model has helped scholars and researchers understand and explain the processes underlying reading aloud across different writing systems including non-alphabetic Chinese. Researchers have argued that skilled native Chinese readers heavily rely on the addressed or lexical route which involves a direct mapping between orthography to phonology (e.g., ref [13,14]). The assembled or non-lexical route, however, is different for Chinese compared to the alphabetic writing systems. Although some sort of non-lexical route is possible in Chinese [15], given the existence of phonetic radicals that are part of many compound characters, the function of phonetic radicals is fundamentally different from that of letters in alphabetic systems [16]. While an alphabetic writing system allows readers to rely on a fully regular letter string for reading aloud a word, the Chinese character is generally considered to be pronounced via retrieving the morpheme that is connected to the spoken language.

In the line of studies that addressed reading aloud in Chinese, previous research has heavily focused on segmental phonology; fewer studies have addressed the independent role of tonal information. There are four tones in Mandarin Chinese. Taking a segmental syllable (e.g., *ma*) in Mandarin Chinese and placing the four tones on it, four different morphemes accompanied by four different meanings are obtained [17]. 妈 (*mā*, tone 1) means *mother*, 麻 (*má*, tone 2) means *hemp*, 马 (*mă*, tone 3) means *horse*, and 骂 (*mà*, tone 4) means *to scold* (hereafter the tone markers are used to denote the tones). Sensitivity to tonal information is not only important for distinguishing speech syllables, but also mapping them to their correct representations in print. In visual word recognition, it has been shown that tone awareness is robust predictor of character recognition in young Chinese readers [18,19]. Given the crucial contribution of tone as a suprasegmental feature in determining the meaning of a Chinese syllable and character, the function of suprasegmental information should also be considered in the theoretical models of reading Chinese aloud.

The relative importance of segmental and tonal information in reading Chinese characters has been examined in a variety of experimental tasks, including silent reading, color naming, conceptually driven spoken word production, and reading aloud. In most studies, the critical manipulation is whether the target and the non-target share the same segmental syllable (S+T-), the same tone (S-T+), or both (S+T+). The accuracy or response times of these critical conditions were compared to the control stimuli that differ from the target in both the segmental syllable and tone (S-T-). Significant effects in both the S+T- and S-T+ conditions would suggest that segmental and tonal information were encoded independently in phonological encoding of characters.

Using a silent reading task in an event-related potential study, Zhang and Damian [20] showed that segmental and tonal information are activated automatically and independent of each other. In a Go/NoGo task, participants were instructed to press a button when the picture names contained the target phonological dimension without overtly producing the response. Results showed that, the onset latencies of N200, a function of neural activity for response inhibition, were earlier when the NoGo trials were contingent on a particular onset (i.e., different onsets but the same rime and tone) than when the NoGo trials were contingent on a particular tone (i.e., S-T+). These results suggest that the segmental information is available for encoding earlier than the tonal information.

In a color naming task (e.g., Stroop paradigm) [21] in which native Mandarin-speaking participants were asked to name the ink colors of Chinese characters, both Li, Lin, Wang, and Jiang [22] and Spinks, Liu, Perfetti, and Tan [23] found significant facilitation effects for both S+T+ and S+T-. The critical stimuli in Spinks et al. included color characters (e.g., 红, *hong2*, *red*), homophones of the color characters (the same syllable and same tone, S+T+, e.g., 洪, *hong2*, *flood*), partial homophones that shared only the same syllable (S+T-, e.g., 轰, *hong1*, *boom*), and the neutral stimuli (S-T-, e.g., 贯, *guan4*, *passing through*) [23]. Li et al. added another stimulus type in order to study the independent contribution of tone: S-T+, partial homophones that shared only the same tone (e.g., 瓶, *ping2*, bottle) [22]. Significant facilitation for congruent S+T+ and S+T- characters was reported in both studies, and for S-T+ characters in Li et al. [22]. Li et al also showed that segmental information played a more important role than tonal information with a stronger facilitation shown for congruent S+T- than S-T+ characters [22].

Using a form preparation paradigm, Chen, Chen and Dell [24] found significant effects for S+T- in spoken word production. Although the process of conceptually driven spoken word production is not the same as that of reading words aloud, both of these processes involve the articulation stage that explicitly requires phonological encoding and vocal responses. Various speech production models [25–28] all agree that segments play an essential role in planning and preparation for speech production. Previous research has provided strong evidence for phoneme segments as the phonological planning unit in languages such as Dutch and English [29–31]. However, in many languages such as Chinese, it has been shown that it is not the phoneme but syllable segment that is selected by speakers for planning spoken words [31–33]. The cued recall task in Chen et al. consisted of two phases. In the learning phase of the study, participants memorized word pairs consisting of a written cue word and a written response word. In the test phase, participants had to produce the response word upon seeing the cue word. Chen et al. showed that native Mandarin speakers benefited from the fore-knowledge of the initial syllable (S) but not the tone (T) when producing disyllabic response words, suggesting that segmental information plays a more important role than tonal information. These results were further replicated in a computer simulation study that showed faster picture naming in the S+T- condition and the absence of priming effect in the S-T+ condition [33]. These results suggest that the syllable is the proximate unit underlying phonological encoding in speech planning and production in Chinese [31] and tone may not be automatically encoded (although studies on Germanic languages showed that stress, another type of suprasegmental information, is encoded in speech production [33,34]).

Significant S+T+ and S+T- effects have also been found in several studies using a reading aloud task. For example, Verdonschot, Lai, Chen, Tamaoka, and Schiller [35] asked participants to name monosyllabic Chinese characters in a masked priming paradigm. Researchers found significant S+T+ facilitation with a 50ms prime duration and a 500ms forward mask. Furthermore, You, Zhang, and Verdonschot [36] observed significant S+T- priming effects with both forward and backward masks (20ms) in a disyllabic Chinese word- naming task. Given the short prime duration and the presence of masks, conscious awareness of the primes was unlikely in both studies. These results suggest the activation of the segmental syllable was automatic and independent of tonal information in reading aloud.

Nixon, Chen, and Schiller used the picture-word interference paradigm (PWI) in which the target picture had a disyllable name with tone sandhi (T3T3, e.g., fŭdăo 辅导 *tutor*) and the distractors were one-character words with the same segmental syllable as the target initial syllable (Experiment 1). Two stimulus onset asynchronies (SOAs) were used (a simultaneous presentation and a delayed presentation of the distractor, that is, SOA = 0ms and 83ms, respectively). When the distractors were delayed, significant facilitation effects were found when there was a match in both tone and segmental syllable between the target picture and the distractor word (S+T+) compared to when there was a match in segmental syllable only (e.g., S+T-). With simultaneous presentation, there was a match in the effect of facilitation between the rising tonal contour (T2) (e.g., fú 服 clothes) and the underlying tone category (T3) (e.g., fŭ 斧 *axe*) compared to control condition with no tone overlap (T1 or T4). This result suggests that there is an activation of multiple-level phonological representations in T3 sandhi production. Wong and Chen [25] used the PWI paradigm to examine the processing of segmental and tonal information in Cantonese with four different SOAs: -200, -100, 0, and 100ms. There was significant facilitation for both S+T+ and S+T- but not S-T+, with stronger effects for S+T+ and there was no significant effect of SOA (Experiment 3). These results indicate that the segmental syllable itself is sufficient to produce a reliable priming effect, although tone alone is not enough to produce a significant effect.

In summary, the S+T+ and S+T- effects have been established in previous studies using tasks involving silent reading, color naming, conceptually driven spoken word production, and reading aloud. However, there are at least two areas of inconsistencies. First, in the two PWI studies, while Wong and Chen [37] found stronger S+T+ effects compared to S+T- effects across all SOAs, Nixon et al. [38] only observed this difference in facilitation effects with delayed presentation of the distractor. In addition, both color naming studies did not show such difference between S+T+ and S+T- effects [22,23]. Second, while Li et al. [22] found a significant S-T+ effect in a color-naming task using the Stroop paradigm, Wong and Chen [37] found tone alone was not sufficient to produce reliable facilitation effect regardless of SOAs in a picture-naming task using the PWI. These inconclusive findings clearly point to the need for further research. The current study aimed to address these inconsistencies by using a primed character reading aloud task with three SOAs (57, 100, and 200ms) and a full factorial design (e.g., S±T±).

### The Current Study

The present study used an unmasked priming paradigm to examine phonological representation and encoding in reading Chinese characters aloud; the prime was only briefly visible and participants were asked to read the target aloud as quickly as possible. Prime duration was manipulated to study the time course of segmental and tonal encoding. Based on Zhang and Damian [20] who found earlier onset latency for segments than tones in a Go/NoGo task, we hypothesized that segmental information may be activated earlier than tonal information. We hypothesized that a facilitative priming effect would be shown for the S+T- stimuli pairs in a shorter prime duration whereas such an effect would only be shown for the S-T+ stimuli in a longer prime duration.

Pinyin is a Roman alphabetic system that transcribes the pronunciations of Chinese characters. All children in the first ten weeks of first grade (6 to 7 years old) in Mainland China are taught to read Pinyin before learning to read and write Chinese characters. Pinyin is a relatively transparent system with two ways to mark the tonal information. One way is to mark the tone explicitly on the vowel using a diacritic [39]. For example, the Pinyin symbol for the character 妈 (mother) can be *mā*, in which the tone diacritic is explicitly marked above the vowel *a*. The second way to represent the tonal information is to use numbers after the segmental information. For example, the Pinyin symbol for the character 妈 (mother) can also be *ma*1, in which the number 1 denotes tone 1. In the present study, we focused on the role of the diacritic representation of tone in tonal processing in Pinyin. The use of Pinyin for reading in a logographic writing system offers an ideal case for testing the relative contribution of segmental versus suprasegmental information for phonological encoding in different writing systems within the same language.

Chen, Fu, Iversen, Smith, and Matthews compared brain activity between character and Pinyin reading in a lexical decision task [24]. In an fMRI study, native Mandarin Chinese speakers were asked to judge whether a visual word written in Pinyin or as a character sounded like a real word. Results showed that different regions were activated when reading Pinyin compared to when reading characters, although overlap also existed. Reading Pinyin led to greater activation in the brain areas responsible for phonological assembly (similar to the areas activated when reading English), whereas reading characters led to greater activation in the areas responsible for visual-orthographic processing. Motivated by these differences in silent reading Pinyin versus characters, the current study investigated the phonological encoding of both segmental and tonal information in Pinyin as compared to characters when serving as primes in a character reading aloud task. Given the alphabetic nature of the Pinyin system and the salient tone marker, we hypothesized that participants would show stronger sensitivity to both segmental and tonal information when they are processing Pinyin primes compared to when they are processing character primes. Furthermore, we examined whether characters are read aloud differently compared to Pinyin in a simple naming task without any primes. Is it possible that the hypothesized stronger sensitivity to segmental and tonal information in processing Pinyin primes stems from the faster reading aloud of Pinyin itself?

The current study was guided by the following questions: 1) Do skilled readers represent and encode both segmental and tonal information for reading aloud characters to the same degree across time? 2) Does the encoding of segmental and tonal information differ between characters and Pinyin? Two unmasked primed naming and a simple naming tasks were conducted. For the two primed naming tasks, the targets were always written characters. The primes were presented as characters in Experiment 1 and as Pinyin in Experiment 2. We used 57 ms, 100 ms, and 200 ms prime durations in both experiments. The selection of the three prime durations was based on Experiment 3 of Zhou and Marslen-Wilson [40] who used the same prime durations to investigate the activation of phonology, orthography, and semantics in reading Chinese characters. To tease apart the processing of segmental and tonal information, we varied the two sources of phonological information in a full factorial design (i.e. S±T±). Note that since that the primes were unmasked in the current study, they were likely to reach conscious processing. For the simple naming task (Experiment 3), we asked the participants to read aloud the character and Pinyin primes used in Experiment 1 and Experiment 2 and compared the speed and accuracy in naming these two different orthographic forms.

## Experiment 1: Character Priming

In Experiment 1, we investigated the encoding of segmental and tonal information in reading aloud while using characters as primes. Since the Chinese writing system is logographic, it is possible that neither segmental nor tonal information in the primes is represented and encoded. However, based on the findings from Spinks et al. [23] and Li et al. [22], it is also possible that both forms of phonological information are encoded, with a stronger effect for segmental than tonal information. With regard to the time course, we hypothesized that longer prime duration would result in stronger phonological facilitation.

### Participants

Participants consisted of 72 native Mandarin speakers, whose ages ranged from 19 to 25 years (*M* = 21.8, *SD* = 1.76). They were randomly assigned to the three prime durations: short (57 ms: 9 males, 14 females), medium (100 ms: 8 males, 16 females), and long (200 ms: 10 males, 15 females). All participants were college students from Beijing Normal University. All participants had normal or corrected-to-normal vision.

### Ethics Statement

The current study was approved by the Institutional Review Board (IRB) of the University of Maryland, College Park, U.S.A. Written consent was obtained from the participants prior to the experiments.

### Materials and Design

Primes and targets were both Chinese characters (See S1 Appendix for stimuli). The prime characters did not share semantic or phonetic radicals with the target characters. Previous research has shown that both semantic and phonetic radicals could provide cues for character identification [41,42]. Ensuring that the primes and targets do not share phonetic or semantic radicals allowed us to examine the phonological processes involved in reading aloud after minimizing the possible confound from orthography. There were 240 prime-target pairs (4 priming conditions × 60 target characters). Presentation of the paired stimuli followed a Latin-square design, in which four lists of prime-target pairs (60 in each list) were created and participants were randomly assigned to each of the lists. The four primes for each target were randomly assigned to each of the four lists (list 1, 2, 3, and 4), so that each target character occurred only once in each list. List was a between-subject variable. For each prime duration group, the participants were randomly assigned to a list. Since prime duration was a between-subject variable, the four lists were exactly the same across the three prime duration groups (i.e., List 1 in the 57ms prime duration group was exactly the same as List 1 in the other two duration groups). There were four types of prime-target pairs: 1) S+T+ (prime and target sharing the same segmental syllable and tone, e.g., 连, *lián*, link—怜, *lián*, pity); 2) S+T- (same segmental syllable, different tone, e.g., 练, *liàn*, practice—怜, *lián*); 3) S-T+ (same tone, different segmental syllable, e.g., 成, *chéng*, become—怜, *lián*); and 4) S-T- (control, e.g., 秀, *xiù*, beautiful-怜, *lián*).

We controlled for character frequency, number of strokes, and number of radical components across the four priming conditions. Polyphone characters, such as 会 which can be pronounced in multiple ways (huì or kuài), were excluded. Characters with phonetic radicals that share the full segmental information with the whole characters, such as 抱 (bào) whose phonetic radical is 包 (bāo), were also excluded to minimize the influence of phonetic radicals on reading aloud. Ten native Chinese speakers, who did not participate in the formal experiment, rated semantic relatedness and another ten rated orthographic similarity of the prime-target pairs, based on a 7-point scale (e.g., for semantic relatedness: 1 = *not related at all* and 7 = *exactly the same meaning*). Only character pairs with an average score ≤ 3 in both rating tasks were selected. See Table 1 for information about the stimulus characteristics and see Supporting Information for a complete list of stimuli. Note that we included similar number of primes in the S-T+ and S-T- conditions that have different syllabic structures (i.e. CV vs. CVV vs. CVC vs. CVVC) from the target characters. There were 37 such pairs for S-T+ and 36 for S-T-.

**Table 1:** Characteristics of the target and prime characters.

	Target	S+T+ Prime	S+T-Prime	S-T+ Prime	S-T- Prime
Frequency	0.25(0.35)	0.56(0.53)	0.56(0.65)	0.59(1.22)	0.57(0.77)
Number of Strokes	8.63(2.63)	7.73(2.29)	7.77(2.28)	8.32(2.32)	7.85(1.97)
Number of Components	2.38(0.88)	2.20(0.79)	2.27(0.75)	2.35(0.87)	2.23(0.69)
Sample Character	怜	连	练	成	秀
Orthographic Similarity between the Prime and Target	N/A	2.025	2.090	2.005	1.925
Semantic Relatedness between the Prime and Target	N/A	1.078	1.065	1.043	1.055
Sample Pinyin	lián	lián	liàn	chéng	xiù
Translation	pity	link	practice	become	beautiful

Note. Standard deviations are in parentheses. Frequency is based on one per thousand counts.

### Procedure

The priming task was implemented using the DMDX software with the following procedure. After initial instructions, a fixation cross (a "+" at the center of the screen) was shown for 500 ms. A priming character then appeared for 57, 100, or 200 ms, followed by the target character, which remained visible until a response was made. The target character was in the same font and size as the prime (48 song-ti). Both the prime and the target were presented at the center of the screen, where the fixation cross was presented. The visual angle was about two degrees horizontally and vertically with the viewing distance at around 60 cm. Participants read the target character aloud into a microphone. Participants were told to ignore the primes (if they were visible). Each participant was randomly assigned to each of the four lists so that each participant only named each target once in the experiment. Participants received five practice trials, during which the experimenter provided feedback. There were 60 test trials and no feedback was given. During the experiment, the second author sat behind the participants and scored their naming accuracy. It took approximately 10 minutes for each participant to complete the task.

### Results and Discussion

Three out of the 60 sets of stimuli were removed from the analyses as one character in each of the three sets contains a phonetic radical that shares full segmental information with the whole character, thus leaving a total of 57 sets of stimuli in the final analyses. All analyses were based on log-transformed data and they did not violate the assumption of normal distribution according to the Mauchly's Test of Sphericity (*χ* ^2^(5) = 7.146, *p* = .210). Analysis of reaction times (Log RTs, hereafter RTs) was based on correct responses only. Failure to trigger the voice key resulted in the removal of 1.5% of the trials. Data above or below two standard deviations (1.7%) from the mean for each condition were removed. Mean RTs, SDs, and error rates for each condition are shown in Table 2. A 3 (prime duration: 57 ms, 100 ms, and 200 ms) × 4 (prime condition: S+T+, S+T-, S-T+, and S-T-) × 4 (list: 1, 2, 3, and 4) mixed ANOVA was conducted separately for RTs and error rates. Prime condition was a within-subject variable and prime duration and list were between-subject variables. The RT analysis showed a significant main effect of prime duration (*F*_*1*_ (2, 60) = 5.473, *p* = .007, *MSe* = .090; *F*_*2*_ (2, 155) = 92.002, *p* < .001, *MSe* = .011) and prime condition (*F*_*1*_ (3, 180) = 8.852, *p* < .001, *MSe* = .001; *F*_*2*_ (3, 465) = 6.767, *p* < .001, *MSe* = .004). All interactions did not reach statistical significance (all *F*s <1). Participants’ RT in the 200ms condition was significantly shorter than that in 57ms (*p* = .003) and 100ms (*p* = .013). The longer prime duration may have provided participants with more time to prepare for and thus speed up the naming of the target characters.

**Table 2:** Mean RTs and error rates in Experiment 1.

Prime Duration	Prime Condition	Example (Prime/Target)	RT (ms)	Error Rates	Priming (ms)
57 ms	S+T+	连/怜	681 (102)	.028 (.046)	16*
	S+T-	练/怜	702 (106)	.050 (.041)	-5
	S-T+	成/怜	692 (100)	.043 (.056)	5
	S-T-	秀/怜	697 (104)	.041 (.056)	
100 ms	S+T+	连/怜	664 (117)	.030 (.039)	20*
	S+T-	练/怜	675 (102)	.036 (.059)	9
	S-T+	成/怜	679 (96)	.025 (.039)	5
	S-T-	秀/怜	684 (104)	.036 (.043)	
200 ms	S+T+	连/怜	590 (82)	.031 (.048)	13*
	S+T-	练/怜	605 (87)	.027 (.038)	2
	S-T+	成/怜	613 (90)	.033 (.050)	-6
	S-T-	秀/怜	607 (83)	.027 (.047)	

*Note*. Standard deviations are in parentheses. The measure for the error rates was out of 100.

*: *p* < .05

Overall, participants’ RTs in the S+T+ condition were significantly shorter than those in each of the other three conditions (all *p*s< .001), whereas the other pairs of conditions did not show any significant difference (*p*s> .10). For the planned comparisons, across all prime durations, participants showed significant facilitation in the S+T+ condition compared to the control condition (57 ms: *t*_*1*_ (22) = 2.105, *p* = .047, *SD* = .046, *t*_*2*_ (56) = 2.474, *p* = .016, *SD* = .089; 100 ms: *t*_*1*_ (23) = 2.631, *p* = .015, *SD* = .062, *t*_*2*_ (55) = 1.431, *p* = .158, *SD* = .127; 200 ms: *t*_*1*_ (24) = 2.171, *p* = .040, *SD* = .068; *t*_*2*_ (56) = 2.295, *p* = .025, *SD* = .097). However, participants did not show any significant facilitation effect in the S+T- or S-T+ condition (all *p*s > .1). Note that in the S+T- condition, it was difficult to find any non-significant trend across the three prime durations (see Table 2). The main effect of list was significant in the item analysis only (*F*_*1*_ < 1; *F*_*2*_ (3, 155) = 3.954, *p* = .009, *MSe* = .042). The significance in the item analysis might be due to some characters with low frequency. Since there were only 60 target characters, it is possible that for some lists, targets with relatively low frequency (e.g., 帚, zhou3, meaning *broom*) happened to have a particular type of prime, such as S+T+, and this facilitation effect is stronger than characters with high frequency since the space for improvement in response latency is larger for the low-frequency characters. This possibility may be tested in future research with the inclusion of more target characters. Analysis of accuracy rates did not yield any significant effect (all *p*s > .10).

The significant facilitative priming effect of the S+T+ pairing across the three prime durations suggests that phonological information is clearly represented and encoded quickly in reading Chinese characters aloud. These results were consistent with those in Verdonschot et al. [35] in the masked priming paradigm and partially consistent with those in Wong and Chen [37] who used the PWI paradigm to examine Cantonese spoken word production and showed faster responses when the picture and word distractor shared the same syllable and tone (Experiment 3).

A significant facilitation effect was not observed with S+T- or S-T+ primes, suggesting that shared segmental syllable or tone alone might not be sufficient to aid character naming. The absence of S-T+ effects is in line with the results in Wong and Chen [37] who observed null effects on naming latencies when the picture name and the distractor shared the same tone but differed in segmental syllable (Experiment 1). However, the absence of S+T- effects was inconsistent with previous research that showed the significant S+T- effects [22,31,33,36,37]. Note that the current study used different stimuli materials and task paradigms compared to the aforementioned studies. For example, Wong and Chen [37] utilized a picture-naming task whereas the current study used a primed character-reading aloud task. Furthermore, Cantonese phonology is different from that of Mandarin and these differences include larger number of tones (six or nine tones according to different ways of categorization), greater syllable coda diversity, different consonant inventory, and vowel backness [43]. These cross-linguistic variations may explain the discrepancy in the results from the current study and those from Wong and Chen [37].

In the Stroop task used in Li et al, the target color and the distractor character were presented simultaneously and the distractor character stayed on the computer screen until a naming response was made. In the priming paradigm used in the current study, however, the prime and target were presented consecutively and the prime was presented only for a brief period of time (57, 100, or 200ms). Perhaps the brief presentation of the prime is not sufficient to allow for separate activation between syllable segmental and tonal information. If longer prime durations were used, independent activation of segmental and tonal information may occur. On the other hand, the facilitation of T2 or T3 distractor (e.g., S+T-) in Nixon et al. was presumably related to the unique characteristics of tone sandhi, which was not involved in the present study. Nixon et al. also differed from the current study in terms of the control condition. The control words in Nixon et al. were always S+T- words which have either T1 or T4 whereas in the current study the control words were always S-T-. Furthermore, in the form preparation paradigm used by Chen et al. and O’Seaghdha et al. [31, 32], production was conceptually driven (i.e., starts from concepts), whereas naming in the current study starts from orthographic encoding. In addition, the response items were not visually presented in the cued recall task, whereas the primes and response targets were both visually presented in the present study. Therefore, we speculated that the logographic orthographic information may have cued participants to process segmental and suprasegmental information as an integral unit, whereas in a conceptually driven production task the two sources of phonological information could be encoded in a parallel but separate fashion.

An important feature of the current experiment is that the targets and primes did not share phonetic or semantic radicals. In addition, we also excluded any characters containing phonetic radicals that share the full segmental information with the whole characters. This control allowed us to tease apart the influence of orthography on the encoding mechanism of segmental and tonal information in reading aloud. Our stringent criteria for stimuli selection may help explain the absence of the S+T- effect in the present study. It is possible that, without the additional orthographic cue for pronunciation, having the same segmental syllable in the prime was not enough to facilitate the naming of the target character.

Overall, the current results appeared to suggest that segmental syllable and tone are likely to be encoded as an integral unit when reading characters without full phonetic radicals in an un-masked primed reading aloud task. In contrast to characters in which no reliable phonological information is represented in the orthography, Pinyin has a transparent phoneme-grapheme correspondence. It is possible that when both segmental and tonal information is represented explicitly in the orthography, as the case of Pinyin, the encoding of both types of information is likely to be facilitated. This possibility was examined in Experiment 2.

## Experiment 2: Pinyin Priming

We hypothesized that readers should be more sensitive to the segmental and tonal information in the target character with the presentation of Pinyin primes than the presentation of character primes given that phonological information is explicitly represented in Pinyin primes. We also hypothesized that segmental information plays a more important role than tonal information in reading aloud characters, similar to the finding shown in Li et al. [22]. Regarding the time course of encoding Pinyin syllables, if the left-to-right serial phonemic computation process [10,11] is involved, it is possible that the segmental information may be encoded earlier than the tonal information in Pinyin primes. Previous studies on the perception of spoken Chinese syllables suggest that tone is tied more closely to vowel than onset [44,45].

### Participants

The participants were the same as those in Experiment 1. The order of the two experiments was counterbalanced between participants. There was a five-minute break between the two experiments.

### Materials and Design

Materials were the same as those in Experiment 1, except that the primes in Experiment 2 were presented in Pinyin. In addition, different primes were assigned to the 60 targets to create four lists that were different from the four lists in Experiment 1.

### Procedure

The procedure of Experiment 2 was exactly the same as that of Experiment 1. Experiment 2 took approximately 10 minutes to complete.

### Results and Discussion

Mean RTs, SDs, and error rates for each condition are shown in Table 3. The procedure of data cleaning was the same as that of Experiment 2. RTs were log-transformed and the transformed data did not violate the assumption of normal distribution according to the Mauchly's Test of Sphericity (*χ* ^2^(5) = 6.456, *p* = .264). Similar to Experiment 1, participants showed a significant main effect of prime condition (*F*_*1*_ (3, 180) = 21.326, *p* < .001, *MSe* = .001; *F*_*2*_ (3, 462) = 17.858, *p <* .001, *MSe* = .005) and prime duration (*F*_*1*_ (2, 60) = 8.655, *p* < .001, *MSe* = .072; *F*_*2*_ (2, 154) = 51.360, *p* < .001, *MSe* = .016). The interaction between prime condition and prime duration was significant in the item analysis (*F*_*1*_ (3, 180) = 1.449, *p* = .198, *MSe* = .001; *F*_*2*_ (6, 462) = 2.151, *p* = .047, *MSe* = .005). The main effect of list was not significant (both *Fs <* 1). The interactions between prime condition and list, prime duration and list, and the three-way interaction were not significant (all *F*s < 1).

**Table 3:** Mean RTs and error rates in Experiment 2.

Prime Duration	Prime Condition	Example (Prime/Target)	RT (ms)	Error Rates	Priming (ms)
57 ms	S+T+	lián/怜	623 (82)	.041 (.056)	23**
	S+T-	liàn/怜	631 (80)	.038 (.052)	15*
	S-T+	chéng/怜	646 (87)	.043 (.047)	0
	S-T-	xiù/怜	646 (82)	.023 (.043)	
100 ms	S+T+	lián/怜	659 (85)	.025 (.033)	42**
	S+T-	liàn/怜	683 (82)	.022 (.032)	18*
	S-T+	chéng/怜	694 (82)	.036 (.043)	7
	S-T-	xiù/怜	701 (109)	.019 (.031)	
200 ms	S+T+	lián/怜	572 (84)	.021 (.046)	29***
	S+T-	liàn/怜	576 (96)	.021 (.042)	25***
	S-T+	chéng/怜	586 (96)	.050 (.053)	15**
	S-T-	xiù/怜	601 (108)	.032 (.048)	

*Note*. Standard deviations are in parentheses. The measure for the error rates was out of 100.

*: *p* < .05

**: *p* < .01

***: *p* < .001

Planned comparisons showed that at 57 ms, participants showed significant facilitation for S+T+ (*t*_*1*_ (22) = 2.890, *p* = .009, *SD* = .061, *t*_*2*_ (55) = 3.252, *p* = .002, *SD* = .082) and S+T- (*t*_*1*_ (22) = 2.307, *p* = .031, *SD* = .051, *t*_*2*_ (55) = 2.187, *p* = .033, *SD* = .082). Similarly, at 100 ms, participants showed significant S+T+ facilitation (*t*_*1*_ (23) = 3.860, *p* < .001, *SD* = .075, *t*_*2*_ (54) = 2.420, *p* = .019, *SD* = .147) and S+T- facilitation in the subject analysis (*t*_*1*_ (23) = 2.075, *p* = .049, *SD* = .063, *t*_*2*_ (54) = 1.214, *p* = .230, *SD* = .154). At 200 ms, participants showed significant facilitation across all priming conditions: S+T+ (*t*_*1*_ (24) = 3.854, *p* < .001, *SD* = .059; *t*_*2*_ (56) = 2.980, *p* = .004, *SD* = .183), S+T- (*t*_*1*_ (24) = 3.764, *p* = .001, *SD* = .056; *t*_*2*_ (56) = 3.494, *p* = .001, *SD* = .161), and S-T+ in the subject analysis (*t*_*1*_ (24) = 3.127, *p* = .005, *SD* = .039; *t*_*2*_ (56) = 1.725, *p* = .090, *SD* = .199).

Across all three prime durations, the effect size for the S+T+ condition was greater than that for S+T-, but the difference only reached significance at 100 ms in the subject analysis (*t*_*1*_ (23) = 4.245, *p* < .001, *SD* = .044; *t*_*2*_ (55) = 1.394, *p* = .169, *SD* = .155). At 57 ms, the effect size for S+T- facilitation was significantly larger than that for S-T+ facilitation in the subject analysis (*t*_*1*_ (22) = 2.800, *p* = .010 *SD* = .039; *t*_*2*_ (56) = 1.539, *p* = .129, *SD* = .102); for 100 ms and 200 ms, this difference did not reach statistical significance (100ms: *t*_*1*_ (23) = 1.526, *p* = .141, *SD* = .046; *t*_*2*_ (54) = .792, *p* = .432, *SD* = .146; 200ms: *t*_*1*_ (24) = .287, *p* = .776, *SD* = .053; *t*_*2*_ (56) = 1.686, *p* = .097, *SD* = .129). Analysis of accuracy rates did not show any significant effect (all *p*s > .1).

These results suggest that, when Pinyin served as primes, segmental and suprasegmental information were likely to be encoded separately. Tone is marked on top of the vowel (e.g., *mā*) in Pinyin; this nonlinear structure may help readers separate segmental and tonal information. The fact that the S-T+ facilitation was only significant at 200 ms prime duration in the subject analysis suggests that tone is likely to be encoded later than segmental information. The consistent S+T- facilitation at both 57ms and 200ms prime durations (and a marginally significant facilitation at 100ms) and a trend of a stronger effect for S+T- than S-T+ suggests that the segmental information plays a more important role in facilitating character naming than the tonal information.

We further compared the data in Experiments 1 and 2 via a 2 (prime type: character vs. Pinyin) x 4 (prime condition: S+T+ vs. S+T- vs. S-T+ vs. S-T-) x 3 (prime duration: 57ms vs. 100ms vs. 200ms) mixed ANOVA. Prime type and prime condition were within-subject factors, and prime duration was a between-subject factor. Significant main effects of prime type and prime condition were found (prime type: *F*_*1*_ (1, 69) = 4.2823, *p* = .042, *MSe* = .039; *F*_*2*_ (1, 162) = 26.648, *p* < .001, *MSe* = .011; prime condition: (*F*_*1*_ (3, 207) = 26.391, *p* < .001, *MSe* = .001; *F*_*2*_ (3, 486) = 12.609, *p* < .001, *MSe* = .007). Participants named characters significantly faster when the prime was Pinyin. A significant main effect of prime duration was also shown (*F*_*1*_ (2, 69) = 8.931, *p* < .001, *MSe* = .118; *F*_*2*_ (2, 162) = 95.928, *p* < .001, *MSe* = .016). Participants’ RTs were shorter with longer prime duration, which is consistent with the separate analyses in Experiments 1 and 2. There were also significant interactions between prime type and prime duration (*F*_*1*_ (2, 69) = 3.220, *p* = .046, *MSe* = .039; *F*_*2*_ (2, 162) = 23.876, *p* < .001, *MSe* = .011) and between prime type and prime condition (*F*_*1*_ (3, 207) = 3.203 *p* = .024, *MSe* = .005; *F*_*2*_ (3,486) = 3.050, *p* = .028, *MSe* = .008). Analysis of the prime duration ×prime type interaction showed that the faster performance for a Pinyin prime compared to a character prime reached significance for the shortest prime duration (57ms) (*F*_*1*_ (1, 69) = 9.811, *p* = .005, *MSe* = .030; *F*_*2*_ (1, 162) = 202.828, *p* < .001, *MSe* = .010). For other prime durations (i.e., 100ms and 200ms), no significant difference was shown between the two prime types (*p*s > .05). Further analysis of the prime type × prime condition interaction showed that the faster performance for a Pinyin prime than a character prime reached significance for the S+T+ and S+T- conditions (all *p*s < .02). For the S-T+ and S-T- conditions, the difference between the two prime types did not reach significance (*p*s > .05). The three-way interaction did not reach significance (*F*_*1*_ (6, 207) = 1.263, *p* = .276, *MSe* = .002; *F*_*2*_ (6, 486) = 1.397, *p* = .214, *MSe* = .008).

Given that the target characters were the same across Experiments 1 and 2, it is possible that participants respond faster (a practice effect) or slower (a fatigue effect) when they saw the target for the second time. As a result, we conducted an analysis using linear mixed effect models [46] to examine the repetition effect. After several forward and backward comparisons, the reduced model (named as Model 1 in short) LogRT ~ PrimeType*PrimeDuration*Condition + (PrimeDuration |Subject) +(Condition |Subject)+ (PrimeDuration |Item) +(Condition|Item) was selected as the final model. When Repetition was added as a single fixed effect (named as Model 2: LogRT ~ PrimeType*PrimeDuration*Condition+ Repetition + (PrimeDuration |Subject) +(Condition |Subject)+ (PrimeDuration |Item) +(Condition|Item), it did not improve the model fit significantly (*χ* ^2^(1) = .317, *p* = .5734). Furthermore, we compared the full model when repetition was added as an interaction term (i.e., LogRT ~ PrimeType*PrimeDuration*Condition*Repetition + (PrimeDuration |Subject) +(Condition |Subject)+ (PrimeDuration |Item) +(Condition|Item) with both Model 1 and Model 2, and neither comparison showed significantly better fit for the full model (both *p*s > .10). Therefore, the Repetition effect (including the main effect and interactions with all other fixed effects) was removed from the subsequent analysis.

Considering that adult Chinese speakers rarely read Pinyin in their daily life, it is remarkable to observe that even within a very short duration (57ms) Pinyin primes produced a stronger facilitation effect compared to character primes and participants’ naming response was significantly faster when the primes were Pinyin than when the primes were characters. Is it possible that the explicit phonological representation in Pinyin allows for more rapid, direct phonological activation in comparison to characters so that the former was read faster than the latter despite Pinyin’s low familiarity? To the best of our knowledge, no previous study has directly compared the simple naming speed of characters and Pinyin in adult Chinese readers. Thus, we carried out Experiment 3 to test this hypothesis.

## Experiment 3: Simple Character vs. Pinyin Naming

It is possible that Pinyin would be named faster than characters as the explicit representation of phonological information in Pinyin may allow for fast and direct phonological activation. Alternatively, Pinyin may be named more slowly compared to characters if readers utilize a phonological assembly route for Pinyin while they utilize a direct, lexical route for naming characters.

### Participants

Twenty native Mandarin-speaking adults from a mid-Atlantic university in the U.S. (10 males, *M*age = 23.2, *SD* = 2.12) who did not participate in the previous experiments took part in Experiment 3. All of the participants were born in Mainland China and have lived in the U.S. for 8 months to 4 years. They all started to learn English at around 9 or 10 years old.

### Materials and Procedure

Materials consisted of all 228 prime characters in Experiment 1 and all 228 Pinyin primes in Experiment 2. All participants completed both the character naming block and Pinyin naming block with the order counterbalanced.

The task was implemented using the DMDX software with the following procedure. After initial instructions, a fixation cross (a "+" at the center of the screen) was shown for 500 ms. Afterwards, the target character or Pinyin was presented for 3000ms or until a response was made. The visual angle was similar to that in Experiments 1 and 2. Participants read the target character/Pinyin aloud into a microphone. It took approximately 20 minutes for each participant to complete the two blocks. The interval between the blocks was about three minutes.

### Results and Discussion

Checkvocal software [47] was used in data scoring. The software simultaneously presented the visual target and the participant’s oral response recorded during the experiment. The experimenter scored accuracy by clicking the “correct” or “wrong” button. If the voice key was not triggered at the onset of oral response, adjustment of the timing was made by clicking the “retrigger” button. All RT data was log-transformed to improve normality. A 2 × 2 mixed ANOVA was conducted with Orthographic Type (character vs. Pinyin) as a within-subject variable and Order (character first or Pinyin first) as a between-subject variable. See Table 4 for the descriptive statistics. There was a significant Orthographic Type main effect, in which Pinyin were named significantly slower (684ms) than characters (588ms) (*F*_1_(1, 18) = 21.425, MSe = .01, *p*< .001; *F*_2_ (1, 227) = 549.131, MSe = .010, *p*< .001). The main effect of Order was significant in the item analysis only (*F*_1_ (1, 18) = 2.7195, MSe = .028, *p* = .117; *F*_2_ (1, 227) = 467.401, MSe = .004, *p*< .001). RTs for the Pinyin-Character order were significantly longer than the Character-Pinyin order. Furthermore, when characters were named first, Pinyin naming seemed to be facilitated. However, when Pinyin was named first, there seemed to a fatigue effect for character naming. Note that the interaction between Orthographic Type and Order was not significant (*F*_1_(1, 18) = .020, MSe = .010, *p* = .888; *F*_2_ (1, 227) = .124, MSe = .004, *p* = .947). In addition, participants’ accuracy rate in character naming (98.9%) is significantly higher than that in Pinyin naming (95.7%) (*F*_1_(1, 18) = 18.753, MSe = .001, *p*< .001; *F*_2_ (1, 227) = 60.006, MSe = .411, *p*< .001). The error types in Pinyin naming included tones, glides, and diphthongs.

**Table 4:** Mean RTs and error rates in Experiment 3.

Order	Orthographic Type	RT(ms)	Error Rates
Character First	Character	562 (68)	.012 (.014)
	Pinyin	652 (87)	.037 (.029)
Pinyin First	Character	614 (53)	.010 (.007)
	Pinyin	716(153)	.053 (.043)

In Experiments 1 and 2, participants’ naming response of characters was faster when primes were Pinyin than when primes were characters. We speculated that this was possibly because of the explicit representation of phonological information in Pinyin led to direct and rapid phonological activation and hence facilitation of naming characters. Results from Experiment 3 raised a question about this possibility since participants took longer to name Pinyin than characters in a simple naming task. This finding could be explained by the Dual-Route Model [10,11]. Naming character might have gone through the direct, whole word, lexical route whereas naming Pinyin used the indirect, letter-sound assembly route. The lexical route is generally faster than the phonological assembly route.

Another possible explanation is that participants may be less familiar with the Pinyin compared to the characters. At the end of the experiment all 20 participants reported that it was more difficult to name Pinyin and it would be much easier if they were allowed to pronounce the Pinyin symbols using the training method taught in early education. Wang and Gao [48] reported that Pinyin is a required course in Mainland China in the first grade. The sequence in which Pinyin was taught is that simple rimes with a single vowel (*a*, *o e*, *i*, *u*, *ü*) were introduced first, together with the four tones, followed by onsets (e.g., *b*, *p*, *m*, *f*). At this point, children were encouraged to blend onsets and vowels together to make open syllables (e.g., *m—ā*→ *mā*). Finally, children learned the compound rimes with vowel diagraphs or nasal coda (e.g., *ing*, *ao*, *ian*, *iang*) and continued practicing blending sounds. Wang and Gao observed a clear rime preference in Grade 1 children tested in Beijing. This instructional approach for reading Pinyin aloud in the early years of schooling may have contributed to the slower naming of a whole Pinyin syllable among the adult participants. Taken together, the explicit phonological information in Pinyin may result in fast activation of onset and rime, yet the need to combine the sub-syllabic units slows down the naming process.

Although participants were slower to name Pinyin as *targets*, they were faster to name the target characters when Pinyin were *primes*. The readers might not have gone through the entire assembly process when processing the Pinyin primes. Instead, they may activate the segmental information, full or partial, without the need to assemble it, in order to facilitate the naming of the target characters. The explicit phonological representation in Pinyin results in faster activation of the segmental information, however, it is the assembly process that makes Pinyin naming more time-consuming than character naming.

## General Discussion

We used an un-masked visual-visual priming paradigm to examine phonological encoding in reading Chinese characters aloud. Our results suggest that in a logographic orthography, segmental and tonal information may be represented and encoded as an integral unit that facilitates character naming. In Pinyin, in which phonological information is explicitly represented, there is separate representation and encoding of segmental and tonal information and tonal information seems to be accessed later.

Previous research has investigated segmental and tonal processing in spoken word recognition in Chinese via the auditory modality. For example, Ye and Connine [49] showed that listeners responded slower in a vowel-tone monitoring task when the item was different from the target only in tone compared to when the items was different only in vowel. In an auditory priming experiment, Lee [50] did not find a significant priming effect when the prime and target only differed in tone (e.g., *lou3* ‘hug’–*lou2* ‘hall’) at both 50ms and 250ms ISIs, suggesting that tonal information was used by Mandarin listeners to distinguish between segmentally identical words. However, when the prime and target were not minimal tone pairs but were related through a third word that was not present in the experiment (e.g., *lou3* ‘hug’–*jian4zhu0* ‘building’, where *jian4zhu0* is semantically related to *lou2* ‘hall’), there was a significant priming effect at 50ms ISI but not at 250ms ISI, suggesting that tonal information is used on-line to reduce the number of possible candidates but does not prevent the minimal tone pairs from being activated until the later phase of lexical activation. In a Go/NoGo task in which participants were asked to withhold response to one type of information, Zhang and Damian [20] showed that the onset latency of the N200 effect was earlier when the response was contingent on the onset consonant as compared to when it was contingent on the tone. These results suggest that tone is encoded later than segmental information in spoken Chinese words. The present study examined the processing of written Chinese words in the Pinyin form and found that tones were activated later than segmental syllables. The time course of tonal activation appears to be consistent across spoken Chinese words and written Pinyin.

The finding that phonological information is represented and encoded in processing both characters and Pinyin is in line with the large volume of literature on fast phonological access in alphabetic writing systems [51–53], and supports the general phonological principle in reading in general across different writing systems [42]. In addition to general phonological representation, the current study suggested that segmental and tonal information may be represented and encoded as an integral unit in a logographic system like Chinese when a set of characters without phonetic and semantic radicals shared between primes and targets were used as stimuli in an un-masked primed naming paradigm. The integration of segmental and tonal information is probably driven by the morpho-syllabic mapping between phonology and orthography in Chinese characters.

With Pinyin primes, participants showed better sensitivity to both segmental and tonal information. This finding suggests that the phonological representation and processing of Pinyin syllables can be separated into segmental and suprasegmental constituents. Pinyin is orthographically transparent—there is a relatively close mapping between phonemes and graphemes. Even though Pinyin may not be a fully-fledged writing system and is hardly read compared to Chinese characters in skilled readers’ daily life, it is used frequently as an input system to type characters on a computer. In addition, Chinese readers often rely on Pinyin to figure out the pronunciations of unfamiliar characters. The finding in Experiment 2 further highlighted the benefit of the explicit phonological representation in Pinyin when facilitating naming characters, even though the speed of naming Pinyin syllables was significantly slower than characters in Experiment 3.

The facilitation by Pinyin primes of target character reading among skilled readers is in line with recent research which showed that Pinyin skills promote character reading in younger children [54]. With Pinyin primes, the trend of stronger facilitation for S+T- than S-T+ across the three prime durations suggests that the segmental information may play a more important role in facilitating character naming than the tonal information. In spoken word recognition, Tong et al. showed that segments played a more robust role than tones [45]. Listeners were asked to classify syllables based only on a target dimension (tone, consonant, or vowel). Results showed that the segmental dimensions interfered more with tone classification than the tonal dimension did with vowel or consonant classification. Tong et al. also suggested that there is greater integrality between tones and vowels than between tones and the initial consonants. Later encoding of tone in our study is likely due to the integrality between tones and vowels, and encoding of tone may not happen until the end of the syllable. The late tone priming effect shown from the Pinyin primes may also be linked to the articulatory preparation needed for reading aloud (but see the recent evidence from ERPs for a rapid activation of tonal information in reading aloud Chinese words [55]). It is important to note that reading aloud entails the articulatory preparation that is impossible without phonological information. It remains unclear whether a similar pattern of results from the current study (i.e., the late tone priming effect) would be shown in a visual recognition task such as a lexical decision task in which detailed phonological information may not be mandatory.

Our findings may be considered as consistent with the well-established Dual-Route Cascade (DRC) model [10,11]. According to the DRC model, there are two possible routes from orthography to phonology. The lexical route assumes that the computation of phonology from print occurs in parallel or simultaneously across the letters. The non-lexical route, on the other hand, assumes that there is a left-to-right serial phonemic computation process across the letters. The lexical route may help explain the findings from Experiment 1 in the current study in which characters served as primes. The encoding of phonology in the characters may occur simultaneously across segmental and suprasegmental information. This may explain the significant effect from only the S+T+ character primes but not the S+T- or S-T+ primes. Another possibility is that tone could still be a later process in character processing. One good case to demonstrate this later process of tone is when tone 3 has to be changed to tone 2 in the context of tone sandhi (e.g., ni3 + hao3 = ni2hao3). We speculated that the change of tone 3 to tone 2 in the first syllable is a sort of post-hoc process that occurs after the preparation of the first and second syllables. In other words, the readers are likely to 1) prepare ni syllable with tone 3, 2) prepare hao syllable with tone 3, 3) change the first ni syllable to tone 2. Empirical research is needed to test this hypothesis.

When Pinyin syllables served as primes, the time course shown in Experiment 2 suggests that there may be a left-to-right serial process that allows the segmental syllable to be encoded first, then tonal information. This may help explain the finding that at the shortest prime duration, the facilitative effect was larger for S+T- Pinyin primes than for S-T+ Pinyin primes whereas at the longer prime durations there was no significant difference in the magnitude of facilitation. Since our naming task entails visual processing as well as naming of the written words, a question may arise as to where tonal activation is located. We suggest that in the processing of Chinese characters, tone is an integral part of the syllable phonology and is likely to be involved lexically toward the end of the visual processing prior to the production of the spoken words. In the processing of Pinyin syllables, on the other hand, tonal activation may occur pre-lexically after segmental activation during visual processing prior to word production. O’Seaghdha et al. [31] and O’Seaghdha [56] made a similar suggestion in terms of pre-lexical activation of segmental syllable prior to word production when explaining their facilitation effect from the atonal syllables (equivalent to our S+T- items) on speech production.

The present study examined the activation of segmental and suprasegmental information in reading Chinese aloud. In both Experiments 1 and 2, onset and rime were treated as a whole unit (i.e., segmental syllable). The time course of activation for onsets and rimes may be different between the characters and Pinyin. While phonological information is not explicitly represented in the characters, phonological information is represented in a linear orthographic structure in Pinyin, with onset being represented first, followed by rime and tone, which is marked on top of the vowel. Future research is needed to further compare the activation of onset and rime in relation to tonal information in processing characters versus Pinyin. Furthermore, one limitation of our stimuli is that six S-T+ primes shared the same rime with the target (填(tian2)-男(nan2), 势(shi4)-治(zhi4), 宛(wan3)-伞 (san3), 援(yuan2)-甜(tian2), 钥(yao4)-到(dao4), 赤(chi4)-既(ji4), three S-T- primes shared the same rime with the target (饰(shi4)-知 (zhi1), 宛(wan3)-钱(qian2) and 限(xian4)-环 (huan2). In addition, one S-T+ prime and one S-T- prime shared the same onsets with the targets (析(xi1)-香(xiang1) and兵(bing1)-表(biao3), respectively). Future research needs to be more careful with the stimuli selection to ensure the cleanest paring of S+T+ vs. S-T+ and S+T+ vs. S-T-. It is also worth noting that in Experiment 2, there is a possibility that the Pinyin primes (such as lian2) might have activated the target character (怜, *pity*), in other words, this could be considered as an identity prime. Future research needs to take this aspect of design into consideration. Finally, we did not use a forward mask in our experiment. The participants may have been conscious about the primes without a mask even at the shortest prime duration (57ms). Hence, the comparison of the current results with those from masked priming studies should be interpreted with caution. Future research may include a forward mask to minimize the visibility of the primes and thus the possibility of processing strategies.

## Conclusion

The current study found novel evidence that in processing Chinese characters, skilled readers may encode the segmental syllable and tone as an integral unit. In contrast, Pinyin may encourage the separation of segmental and suprasegmental information during phonological encoding, with likely later access to tonal information. We speculate that this may be due to the explicit representation of phonological information in Pinyin, although Pinyin is read more slowly than characters. Our findings further point to the need to consider the contributions of both segmental and suprasegmental information and the time course of activation in the well-established word reading models [10,57].

## Supporting Information

S1 Appendix List of target characters and four types of prime-target pairs. (DOCX)

S1 Dataset Raw data from Experiment 1 and Experiment 2. (XLSX)

## References

[1] Bowey JA, Muller D. Phonological recoding and rapid orthographic learning in third-graders’ silent reading: a critical test of the self-teaching hypothesis. J Exp Child Psychol. 2005;92: 203–219. 16095604 10.1016/j.jecp.2005.06.005

[2] De Jong PF, Bitter DJL, van Setten M, Marinus E. Does phonological recoding occur during silent reading, and is it necessary for orthographic learning? J Exp Child Psychol. 2009;104: 267–282. 10.1016/j.jecp.2009.06.002 19608198

[3] Shu H, Chen X, Anderson RC, Wu N, Xuan Y. Properties of school Chinese: Implications for learning to read. Child Dev. 2003;74: 27–47. 12625434 10.1111/1467-8624.00519

[4] Fu D, Zhou J, Zhu WS, Manley PW, Wang YK, Hood T, et al Imaging the intracellular distribution of tyrosine kinase inhibitors in living cells with quantitative hyperspectral stimulated Raman scattering. Nat Chem. 2014; advance online publication. 10.1038/nchem.1961 PMC4205760 24950332

[5] Perfetti CA, Tan LH. The time course of graphic, phonological, and semantic activation in Chinese character identification. J Exp Psychol Learn Mem Cogn. 1998;24: 101–118.

[6] Pollatsek A, Tan LH, Rayner K. The role of phonological codes in integrating information across saccadic eye movements in Chinese character identification. J Exp Psychol Hum Percept Perform. 2000;26: 607–633. 10811166 10.1037//0096-1523.26.2.607

[7] Zhou X, Marslen-Wilson WD. Pseudohomophone effects in processing Chinese compound words. Lang Cogn Process. 2009;24: 1009–1038. 10.1080/01690960802174514

[8] Crystal D. Dictionary of Linguistics and Phonetics. John Wiley & Sons; 2011.

[9] Ashby J, Clifton C Jr. The prosodic property of lexical stress affects eye movements during silent reading. Cognition. 2005;96: B89–B100. 10.1016/j.cognition.2004.12.006 15913592 PMC1479854

[10] Coltheart M, Rastle K, Perry C, Langdon R, Ziegler J. DRC: a dual route cascaded model of visual word recognition and reading aloud. Psychol Rev. 2001;108: 204–256. 11212628 10.1037/0033-295x.108.1.204

[11] Coltheart M, Curtis B, Atkins P, Haller M. Models of reading aloud: Dual-route and parallel-distributed-processing approaches. Psychol Rev. 1993;100: 589–608. 10.1037/0033-295X.100.4.589

[12] Coltheart M. Modeling Reading: The Dual-Route Approach In: Snowling MJ, Hulme C, editors. The science of reading: A handbook. Malden: Blackwell Publishing; 2005 pp. 6–23.

[13] Chen H-C, Shu H. Lexical activation during the recognition of Chinese characters: Evidence against early phonological activation. Psychon Bull Rev. 2001;8: 511–518. 10.3758/BF03196186 11700902

[14] Abramowitz LK, Bartolomei MS. Genomic imprinting: recognition and marking of imprinted loci. Curr Opin Genet Dev. 2012;22: 72–78. 10.1016/j.gde.2011.12.001 22195775 PMC3314145

[15] Seidenberg MS. The time course of phonological code activation in two writing systems. Cognition. 1985;19: 1–30. 10.1016/0010-0277(85)90029-0 4039640

[16] Perfetti CA, Liu Y, Tan LH. The Lexical Constituency Model: Some Implications of Research on Chinese for General Theories of Reading. Psychol Rev. 2005;112: 43–59. 10.1037/0033-295X.112.1.43 15631587

[17] Li CN, Thompson SA. Mandarin Chinese: A Functional Reference Grammar. University of California Press; 1989.

[18] Shu H, Peng H, McBride-Chang C. Phonological awareness in young Chinese children. Dev Sci. 2008;11: 171–181. 10.1111/j.1467-7687.2007.00654.x 18171377

[19] McBride-Chang C, Tong X, Shu H, Wong AM-Y, Leung K, Tardif T. Syllable, Phoneme, and Tone: Psycholinguistic Units in Early Chinese and English Word Recognition. Sci Stud Read. 2008;12: 171–194. 10.1080/10888430801917290

[20] Zhang Q, Damian MF. The time course of segment and tone encoding in Chinese spoken production: an event-related potential study. Neuroscience. 2009;163: 252–265. 10.1016/j.neuroscience.2009.06.015 19524018

[21] Stroop JR. Studies of interference in serial verbal reactions. J Exp Psychol. 1935;18: 643–662. 10.1037/h0054651

[22] Li C, Lin CY, Wang M, Jiang N. The activation of segmental and tonal information in visual word recognition. Psychon Bull Rev. 2013;20: 773–779. 10.3758/s13423-013-0395-2 23400856

[23] Spinks JA, Liu Y, Perfetti CA, Tan LH. Reading Chinese characters for meaning: the role of phonological information. Cognition. 2000;76: B1–B11. 10.1016/S0010-0277(00)00072-X 10822044

[24] Chen Y, Fu S, Iversen SD, Smith SM, Matthews PM. Testing for dual brain processing routes in reading: a direct contrast of chinese character and pinyin reading using FMRI. J Cogn Neurosci. 2002;14: 1088–1098. 10.1162/089892902320474535 12419131

[25] Caramazza A. How Many Levels of Processing Are There in Lexical Access? Cogn Neuropsychol. 1997;14: 177–208. 10.1080/026432997381664

[26] Dell GS. A spreading-activation theory of retrieval in sentence production. Psychol Rev. 1986;93: 283–321. 10.1037/0033-295X.93.3.283 3749399

[27] Levelt WJ, Roelofs A, Meyer AS. A theory of lexical access in speech production. Behav Brain Sci. 1999;22: 1–38. 11301520 10.1017/s0140525x99001776

[28] Roelofs A. Modeling of phonological encoding in spoken word production: From Germanic languages to Mandarin Chinese and Japanese. Jpn Psychol Res. 2014; n/a–n/a. 10.1111/jpr.12050

[29] Meyer AS. The time course of phonological encoding in language production: The encoding of successive syllables of a word. J Mem Lang. 1990;29: 524–545. 10.1016/0749-596X(90)90050-A

[30] Meyer AS. The time course of phonological encoding in language production: Phonological encoding inside a syllable. J Mem Lang. 1991;30: 69–89. 10.1016/0749-596X(91)90011-8

[31] O’Seaghdha PG, Chen J-Y, Chen T-M. Proximate units in word production: Phonological encoding begins with syllables in Mandarin Chinese but with segments in English. Cognition. 2010;115: 282–302. 10.1016/j.cognition.2010.01.001 20149354 PMC2854551

[32] Chen T-M, Chen J-Y. The syllable as the proximate unit in Mandarin Chinese word production: An intrinsic or accidental property of the production system? Psychon Bull Rev. 2013;20: 154–162. 10.3758/s13423-012-0326-7 23065764

[33] Chen J-Y, Chen T-M, Dell GS. Word-Form Encoding in Mandarin Chinese as Assessed by the Implicit Priming Task. J Mem Lang. 2002;46: 751–781. 10.1006/jmla.2001.2825

[34] Schiller NO. Lexical stress encoding in single word production estimated by event-related brain potentials. Brain Res. 2006;1112: 201–212. 10.1016/j.brainres.2006.07.027 16893534

[35] Verdonschot RG, Lai J, Chen F, Tamaoka K, Schiller NO. Constructing initial phonology in Mandarin Chinese: Syllabic or subsyllabic? A masked priming investigation. Jpn Psychol Res. 2014; n/a–n/a. 10.1111/jpr.12064

[36] You W, Zhang Q, Verdonschot RG. Masked syllable priming effects in word and picture naming in Chinese. PloS One. 2012;7: e46595 10.1371/journal.pone.0046595 23056360 PMC3466322

[37] Wong AW-K, Chen H-C. Processing segmental and prosodic information in Cantonese word production. J Exp Psychol Learn Mem Cogn. 2008;34: 1172–1190. 10.1037/a0013000 18763899

[38] Nixon JS, Chen Y, Schiller NO. Multi-level processing of phonetic variants in speech production and visual word processing: evidence from Mandarin lexical tones. Lang Cogn Neurosci. 2014;0: 1–15. 10.1080/23273798.2014.942326

[39] Hanley JR. Learning to read in Chinese The science of reading, a handbook. Oxford, UK: Blackwell; 2005 pp. 296–315.

[40] Zhou X, Marslen-Wilson W. Phonology, Orthography, and Semantic Activation in Reading Chinese. J Mem Lang. 1999;41: 579–606. 10.1006/jmla.1999.2663

[41] Feldman LB, Siok WWT. Semantic Radicals Contribute to the Visual Identification of Chinese Characters. J Mem Lang. 1999;40: 559–576. 10.1006/jmla.1998.2629

[42] Perfetti CA, Zhang S. Phonological processes in reading Chinese characters. J Exp Psychol Learn Mem Cogn. 1991;17: 633–643. 10.1037/0278-7393.17.4.633

[43] Bauer RS, Benedict PK. Modern Cantonese Phonology. Walter de Gruyter; 1997.

[44] Lin CY, Wang M, Shu H. The processing of lexical tones by young Chinese children. J Child Lang. 2013;40: 885–899. 10.1017/S0305000912000281 22849795

[45] Tong Y, Francis AL, Gandour JT. Processing dependencies between segmental and suprasegmental features in Mandarin Chinese. Lang Cogn Process. 2008;23: 689–708. 10.1080/01690960701728261

[46] Bates D, Maechler M, Bolker B, Walker S, Christensen RHB, Singmann H, et al. lme4: Linear Mixed-Effects Models using “Eigen” and S4 [Internet]. 2015. Available: https://cran.r-project.org/web/packages/lme4/index.html.

[47] Protopapas A. Check Vocal: A program to facilitate checking the accuracy and response time of vocal responses from DMDX. Behav Res Methods. 2007;39: 859–862. 10.3758/BF03192979 18183901

[48] Wang M, Gao W. Subsyllabic unit preference in learning to read pinyin syllables. Contemp Educ Psychol. 2011;36: 142–151. 10.1016/j.cedpsych.2011.02.001

[49] Ye Y, Connine CM. Processing Spoken Chinese: The Role of Tone Information. Lang Cogn Process. 1999;14: 609–630. 10.1080/016909699386202

[50] Lee C-Y. Does horse activate mother? Processing lexical tone in form priming. Lang Speech. 2007;50: 101–123. 17518105 10.1177/00238309070500010501

[51] Grainger J, Ferrand L. Phonology and Orthography in Visual Word Recognition: Effects of Masked Homophone Primes. J Mem Lang. 1994;33: 218–233. 10.1006/jmla.1994.1011

[52] Perfetti CA, Bell LC, Delaney SM. Automatic (prelexical) phonetic activation in silent word reading: Evidence from backward masking. J Mem Lang. 1988;27: 59–70.

[53] Schiller NO. Phonology and orthography in reading aloud. Psychon Bull Rev. 2007;14: 460–465. 17874588 10.3758/bf03194089

[54] Lin D, McBride-Chang C, Shu H, Zhang Y, Li H, Zhang J, et al Small wins big: analytic pinyin skills promote Chinese word reading. Psychol Sci. 2010;21: 1117–1122. 10.1177/0956797610375447 20581343

[55] Zovo K, Helk E, Karafin A, Tõugu V, Palumaa P. Label-Free High-Throughput Screening Assay for Inhibitors of Alzheimer’s Amyloid-β Peptide Aggregation Based on MALDI MS. Anal Chem. 2010;82: 8558–8565. 10.1021/ac101583q 20857910

[56] O’Séaghdha PG. Across the great divide: Proximate units at the lexical-phonological interface. Jpn Psychol Res. 2015;57: 4–21. 10.1111/jpr.12074

[57] Harm MW, Seidenberg MS. Are there orthographic impairments in phonological dyslexia? Cogn Neuropsychol. 2001;18: 71–92. 10.1080/02643290125986 20945207

