# Supplementary material for: The Contributions of Segmental and Suprasegmental Information in Reading Chinese Characters Aloud
Source: PLoS One. 2015 Nov 9;10(11):e0142060. doi: 10.1371/journal.pone.0142060 (PMC4638349; doi:10.1371/journal.pone.0142060)
Supplement: S1 Appendix — (DOCX) [file pone.0142060.s001.docx]

# Supporting Information

Appendix: List of target characters and four types of paired primes.

| Target |  | S+T+ |  | S+T- |  | S-T+ |  | S-T- |  |
| --- | --- | --- | --- | --- | --- | --- | --- | --- | --- |
| 户 | hù | 互 | hù | 乎 | hū | 叹 | tàn | 它 | tā |
| 食 | shí | 拾 | shí | 室 | shì | 昨 | zuó | 渐 | jiàn |
| 尾 | wěi | 委 | wěi | 胃 | wèi | 抢 | qiăng | 其 | qí |
| 宛 | wăn | 晚 | wăn | 弯 | wān | 伞 | săn | 钱 | qián |
| 制 | zhì | 质 | zhì | 知 | zhī | 要 | yào | 网 | wăng |
| 彭 | péng | 朋 | péng | 捧 | pěng | 斜 | xié | 坚 | jiān |
| 剂 | jì | 季 | jì | 鸡 | jī | 这 | zhè | 封 | fēng |
| 寺 | sì | 四 | sì | 丝 | sī | 块 | kuài | 扬 | yáng |
| 盐 | yán | 言 | yán | 烟 | yān | 形 | xíng | 阵 | zhèn |
| 梭 | suō | 缩 | suō | 所 | suŏ | 忽 | hū | 象 | xiàng |
| 需 | xū | 虚 | xū | 续 | xù | 轻 | qīng | 捧 | pěng |
| 其 | qí | 齐 | qí | 气 | qì | 阳 | yáng | 互 | hù |
| 帚 | zhŏu | 肘 | zhŏu | 周 | zhōu | 跑 | păo | 阳 | yáng |
| 封 | fēng | 峰 | fēng | 凤 | fèng | 息 | xī | 明 | míng |
| 权 | quán | 全 | quán | 劝 | quàn | 存 | cún | 类 | lèi |
| 焦 | jiāo | 娇 | jiāo | 角 | jiăo | 疏 | shū | 洗 | xĭ |
| 势 | shì | 室 | shì | 实 | shí | 治 | zhì | 春 | chūn |
| 虱 | shī | 师 | shī | 侍 | shì | 哥 | gē | 何 | hé |
| 礼 | lĭ | 李 | lĭ | 丽 | lì | 走 | zŏu | 并 | bìng |
| 幸 | xìng | 性 | xìng | 形 | xíng | 送 | sòng | 光 | guāng |
| 恋 | liàn | 炼 | liàn | 脸 | liăn | 阅 | yuè | 陡 | dŏu |
| 际 | jì | 计 | jì | 击 | jī | 克 | kè | 买 | măi |
| 丝 | sī | 司 | sī | 四 | sì | 她 | tā | 古 | gŭ |
| 限 | xiàn | 县 | xiàn | 弦 | xián | 若 | ruò | 环 | huán |
| 域 | yù | 浴 | yù | 语 | yŭ | 趁 | chèn | 勃 | bó |
| 支 | zhī | 之 | zhī | 止 | zhĭ | 公 | gōng | 无 | wú |
| 斤 | jīn | 巾 | jīn | 进 | jìn | 升 | shēng | 宁 | níng |
| 钥 | yào | 药 | yào | 咬 | yăo | 到 | dào | 思 | sī |
| 羞 | xiū | 修 | xiū | 秀 | xiù | 聪 | cōng | 条 | tiáo |
| 勿 | wù | 务 | wù | 武 | wŭ | 寿 | shòu | 歹 | dăi |
| 延 | yán | 严 | yán | 烟 | yān | 服 | fú | 思 | sī |
| 昔 | xī | 吸 | xī | 戏 | xì | 班 | bān | 摇 | yáo |
| 疏 | shū | 舒 | shū | 竖 | shù | 争 | zhēng | 百 | băi |
| 祸 | huò | 或 | huò | 活 | huó | 办 | bàn | 时 | shí |
| 饰 | shì | 室 | shì | 时 | shí | 就 | jiù | 知 | zhī |
| 析 | xī | 西 | xī | 戏 | xì | 香 | xiāng | 冷 | lěng |
| 枕 | zhěn | 诊 | zhěn | 珍 | zhēn | 股 | gŭ | 庞 | páng |
| 县 | xiàn | 线 | xiàn | 先 | xiān | 页 | yè | 林 | lín |
| 汁 | zhī | 知 | zhī | 直 | zhí | 夸 | kuā | 扫 | săo |
| 器 | qì | 泣 | qì | 起 | qĭ | 课 | kè | 割 | gē |
| 卓 | zhuó | 浊 | zhuó | 捉 | zhuō | 陈 | chén | 竖 | shù |
| 毒 | dú | 独 | dú | 妒 | dù | 伐 | fá | 乖 | guāi |
| 戒 | jiè | 借 | jiè | 姐 | jiě | 射 | shè | 美 | měi |
| 援 | yuán | 原 | yuán | 院 | yuàn | 甜 | tián | 老 | lăo |
| 谅 | liàng | 亮 | liàng | 梁 | liáng | 盼 | pàn | 啄 | zhuó |
| 兽 | shòu | 寿 | shòu | 收 | shōu | 脆 | cuì | 陈 | chén |
| 赤 | chì | 斥 | chì | 吃 | chī | 既 | jì | 经 | jīng |
| 适 | shì | 室 | shì | 拾 | shí | 冒 | mào | 洒 | să |
| 填 | tián | 甜 | tián | 天 | tiān | 男 | nán | 抄 | chāo |
| 额 | é | 鹅 | é | 饿 | è | 雷 | léi | 秋 | qiū |
| 恕 | shù | 树 | shù | 叔 | shū | 顿 | dùn | 宫 | gōng |
| 杰 | jié | 节 | jié | 姐 | jiě | 舌 | shé | 肉 | ròu |
| 竟 | jìng | 净 | jìng | 精 | jīng | 淡 | dàn | 纯 | chún |
| 兵 | bīng | 冰 | bīng | 并 | bìng | 朱 | zhū | 表 | biăo |
| 怜 | lián | 连 | lián | 练 | liàn | 成 | chéng | 秀 | xiù |
| 役 | yì | 亦 | yì | 衣 | yī | 店 | diàn | 罕 | hăn |
| 具 | jù | 句 | jù | 局 | jú | 视 | shì | 秋 | qiū |
